# Bio-Based Polymers with Potential for Biodegradability

**DOI:** 10.3390/polym8070262

**Published:** 2016-07-14

**Authors:** Thomas F. Garrison, Amanda Murawski, Rafael L. Quirino

**Affiliations:** 1 Department of Chemistry, King Fahd University of Petroleum & Minerals, Dhahran 31261, Saudi Arabia; thomasg@kfupm.edu.sa; 2 Department of Chemistry, Georgia Southern University, Statesboro, GA 30460, USA; am02837@georgiasouthern.edu

**Keywords:** bio-based polymers, biodegradability, PLA, vegetable oil-based polymers

## Abstract

A variety of renewable starting materials, such as sugars and polysaccharides, vegetable oils, lignin, pine resin derivatives, and proteins, have so far been investigated for the preparation of bio-based polymers. Among the various sources of bio-based feedstock, vegetable oils are one of the most widely used starting materials in the polymer industry due to their easy availability, low toxicity, and relative low cost. Another bio-based plastic of great interest is poly(lactic acid) (PLA), widely used in multiple commercial applications nowadays. There is an intrinsic expectation that bio-based polymers are also biodegradable, but in reality there is no guarantee that polymers prepared from biorenewable feedstock exhibit significant or relevant biodegradability. Biodegradability studies are therefore crucial in order to assess the long-term environmental impact of such materials. This review presents a brief overview of the different classes of bio-based polymers, with a strong focus on vegetable oil-derived resins and PLA. An entire section is dedicated to a discussion of the literature addressing the biodegradability of bio-based polymers.

## 1. Introduction

Polymeric materials can be classified into either thermoplastics or thermosetting polymers. Thermoplastic polymers consist of well-packed, non-covalently bound polymer chains that can melt and flow when heated above the polymer’s melting point, while thermosetting polymers consist of networks of polymer chains interconnected through covalent bonds. The latter structures do not melt when heated, and cannot be dissolved in a solvent. These differences between thermoplastic and thermosetting polymers have a direct impact on polymer recyclability. It is well accepted that thermoplastics can be easily melted and re-processed during recycling, whereas thermosetting polymers require harsher conditions in order to be converted into other value-added products. Because of its crosslinked chemical structure, thermosetting polymers often exhibit superior mechanical properties, making their breaking down more challenging than that of thermoplastics. Naturally, such aspect is also reflected on the thermal stability of thermoplastics and thermosetting polymers. It is therefore expected that thermosetting polymers be physically harder to degrade than thermoplastics.

The versatility of thermosetting polymers lies in the possibility of easily adjusting their properties by simply changing crosslink density without a need for modification of the overall chemical structure [1]. Most thermoplastics and thermosetting resins in current industrial use are derived from petroleum, imposing a limitation to the polymer industry due to the continuous depletion of crude oil, frequent oscillation in oil price, and various environmental concerns with sustainability, gas emissions, disposal, and recyclability [2,3]. In this context, increasing efforts have been made to date in designing polymeric materials from renewable resources [4].

A variety of renewable starting materials, such as sugars and polysaccharides, vegetable oils, lignin, pine resin derivatives, and proteins, have so far been investigated for the preparation of bio-based polymers [5,6]. Among the various sources of bio-based feedstock, vegetable oils are the most widely used starting materials in the polymer industry due to their easy availability, low toxicity, and relative low cost [4]. Since the year 2000, the chemical industry alone was responsible for consuming more than 15% of the yearly global production of vegetable oils [7]. Unsaturated vegetable oils have been used in the formulation of paints and coatings due to their ability to react with molecular oxygen in the air and form cross-linked materials [8,9]. A variety of thermosetting polymers obtained from the reaction of carbon-carbon double bonds in the fatty acid chains of vegetable oils have been proposed to date [10].

Despite the lack of sufficient literature on the biodegradability properties of vegetable oil-based thermosetting resins, the possibility of cross-linking the carbon–carbon double bonds in polyunsaturated oils through free radical [11], or cationic [12] polymerizations makes the design of bio-based thermosetting polymers from vegetable oils an interesting alternative to petroleum-derived materials. Other approaches for the preparation of vegetable oil-based polymers involve more significant structural changes, such as grafting of acrylate groups for free radical polymerization [13], epoxidation of carbon–carbon double bonds followed by cure with various nucleophiles [14], acyclic diene metathesis polymerization (ADMET) [15], and ring-opening metathesis polymerization (ROMP) [16]. Finally, polyesters, one of the main classes of biodegradable polymers, and polyurethanes have been prepared from the reaction of vegetable oil-based polyols with anhydrides [17], diacids [17], or diisocyanates [18].

By definition, biodegradability consists in the breakdown of matter as the result of the activity of microorganisms, such as fungi and bacteria, which typically secrete enzymes that cleave specific chemical bonds or perform very specific chemical reactions, leading to lower molecular weight products that can then be used in other processes, by other organisms [19]. For the same reasons previously exposed, one would expect regular thermosetting polymers to be less biodegradable than thermoplastics. However, because enzymes act on very specific chemical bonds, it is possible to design polymeric materials that can be broken down by microorganisms. Indeed, in the US and Canada, microorganisms can be genetically modified in order to produce enzymes tailored to cleave desired chemical bonds in existing polymers. Such an approach is currently prohibited in Europe, where only transglutaminases can be used.

There is an intrinsic expectation that novel polymers designed from renewable, and often biodegradable resources, are also biodegradable. Although this is a logical assumption, there is no guarantee that such polymers can be fully biodegradable. Changes in functional groups, crosslink density, and copolymerization with non-biodegradable co-monomers can lead to materials that do not necessarily exhibit significant or relevant biodegradability. Therefore, biodegradability studies are crucial in order to assess the long-term environmental impact of bio-based materials. A study comparing the degradability time of different bio-based systems would be of great interest to a broad audience, and such a research endeavor should definitely be undertaken in a consistent manner in the near future, as bio-based plastics become increasingly popular. Currently, however, the lack of consistency in biodegradability studies, with varying conditions and protocols being applied to different systems, makes it extremely hard and controversial to compare the biodegradability of plastics in general. As an example, 84% mineralization of poly(lactic acid) (PLA) was observed after 58 days under simulated compost conditions [20], whereas poly(ethylene terephthalate) (PET) takes approximately one year to break down into monomers and oligomers when exposed to intense UV irradiation in the environment. The scope of this review is on bio-based materials developed over the past decade for which biodegradability information is available. The review starts by covering different classes of bio-based polymers, with a strong focus on vegetable oil-derived resins due to their versatility and the authors’ field of expertise. Secondly, an entire section is dedicated to PLA. PLA is one of the most popular bio-based plastics and finds wide industrial use nowadays. Finally, the available literature on the biodegradability of bio-based systems is discussed in detail.

## 2. Bio-Based Polymers

When compared to petroleum-based compounds, the use of natural starting materials for the preparation of bio-based products may result in materials with similar and sometimes possibly improved properties [21]. Therefore, new bio-based materials represent a strategic approach for limiting environmental concern while meeting the current demand for polymers and composites. In the discussion of bio-based materials, one topic of great importance is biodegradability. It is worth noting that it is possible to envision the design of bio-based materials with varying rates of degradation in the environment, depending on their application. For example, a more durable material is desirable for structural applications, while it is preferable for disposable goods to be readily biodegradable. Various materials can be prepared from bio-based resources [22], such as soybean and corn protein-based biopolymers [23,24], bio-based paints from vegetable oils [25], biocoatings [26], polyurethane resins [27], polyester amides [28], thermosetting polyolefins [4,29], and cyanate ester resins [30].

### 2.1. Vegetable Oils

Over the past decade, various polymeric systems have been developed based on the crosslinking of vegetable oils through free radical or cationic polymerization reactions [12,31]. It has been observed that oils bearing conjugated carbon-carbon double bonds are more reactive towards polymerization [32]. More recently, ring-opening metathesis polymerization (ROMP) and acyclic diene metathesis polymerization (ADMET) have also been used in the synthesis of vegetable oil-based polymeric materials [16,33]. In a more elaborate approach, the modification of soybean oil with acrylate groups resulted in acrylated epoxidized soybean oil (AESO), which was crosslinked with styrene and reinforced with multi-walled carbon nanotubes and soot [34]. Poly(AESO)-*co*-polystyrene has been reinforced with flax fibers to demonstrate the action of butyrated lignin as a compatibilizer [35]. Similarly, red oak leaves were introduced onto a commercial maleated AESO (MAESO)-based resin resulting in bio-based composites [36].

In the preparation of bio-based cationic composites, glass fibers were added to a resin composed of corn oil (COR), styrene (ST), and divinylbenzene (DVB), with crosslink density and the overall material’s properties being directly dependent on DVB content [37]. It has also been observed that replacement of COR with conjugated corn oil (CCO) resulted in improved properties [37]. Similar cationic thermosetting resins have also been prepared from conjugated soybean oil (CSO) and conjugated low-saturation soybean oil (CLS) [38]. When reinforcing these resins with ligno-cellulosic materials, a switch from cationic to free radical cure was necessary to avoid quenching of the cationic initiator by the polar reinforcement. When these composites are compression molded, the presence of the natural filler helps to minimize shrinkage and prevent extended cracking of the piece [39]. It was also observed that substitution of *n*-butyl methacrylate (BMA) and DVB with dicyclopentadiene (DCPD) results in lower mechanical properties and micro phase separation [39].

Recent improvements in the mechanical properties of free radical bio-based composites have been achieved upon use of maleic anhydride as a compatibilizer [40,41]. Evaluation of the resin composition revealed that conjugated linseed oil (CLO)-based resins exhibit better properties than CSO-based resins due to their higher number of unsaturations [40]. Similar results have been obtained with cationic resins [42]. With a different approach, the thermoset obtained from the ROMP of DCPD and Dilulin^®^ was reinforced with glass fibers [43]. Polyurethane coatings have also been prepared from vegetable oils [44]. As a matter of fact, bio-based polyols can react with diisocyanates to result in polyurethane dispersions (PUDs) [45]. Castor oil and ricinoleic acid have been used as starting materials for the preparation of PUDs [46,47]. Sunflower, canola, soybean, corn, and linseed oils-based polyols have been used for the synthesis of polyurethanes [18]. Methoxylated soybean oil polyols (MSOLs) [48], and castor oil-, MSOL-, and AESO-based polyols have been employed in the synthesis of anionic PUDs [26]. Protonation of amine groups in anionic PUDs results in cationic PUDs with high adhesion to leather and glass [26]. These materials are also antimicrobial, being intentionally designed to be less susceptible to microbial activity [49].

Improvement of anionic PUDs’ mechanical properties has been obtained through copolymerization with acrylates, resulting in bio-based hybrid latexes [50,51]. Surfactant-free, core-shell hybrid latexes were prepared from soybean oil [52]. Other vegetable oil-based systems include polyesters [53] and polyamides [54,55]. The reaction of Nahar seed oil monoglyceride with anhydrides resulted in polyesters for use in composite applications [17]. In more recent developments, fatty amide diols and castor oil amide-based α,ω-dienes, were utilized in the preparation of polyamides by esterification and ADMET polymerization, respectively [54,55].

### 2.2. Cashew Nut Shell Liquid

The liquid extracted from cashew nut shells (CNSL) is a mixture of phenols [56], with the most important constituents being anacardic acid, cardanol, and cardol [57]. High temperature processing of CNSL converts anacardic acid into cardanol [58]. CNSL finds use in flame-retardant applications due to its chemical structure, which includes an aromatic ring [59]. Other applications include synthetic polymers [57,60,61], resole and novolac resins [62], free radical and ionic thermosets [63,64], and novel CNSL-formaldehyde resins [65]. Overall, CNSL-formaldehyde resins prepared by various ways have shown inferior mechanical properties [66]. It has been shown that an optimum performance is obtained for resins with a composition of 40 wt % cardanol [62]. Blends of CNSL-based and conventional resins have also been studied. Novalac resins from cardanol have been used in epoxy resin formulations with higher tensile strength, toughness, and water resistance [67]. Currently, there is no evidence that CNSL-based materials are biodegradable. Their aromatic content and crosslink density are features that may compromise their biodegradability potential.

One of the applications of CNSL outside of the realm of strictly biodegradable materials is the synthesis of cardanol-based polybenzoxazines [68,69] with good thermal and flame retardancy properties [70,71], which can also compromise the biodegradability of these materials. Due to their versatile molecular design, different alternatives have been developed for the preparation of CNSL-based polybenzoxazines, including blending with bisphenol A-based polybenzoxazines [72]. Other studies have reported the use of furfural-modified cardanol [73,74], or cardanol-modified bisphenol A-based benzoxazines [75,76] in order to enhance the performance of these resins. Alternatively, different amines, such as aniline [72], and ammonia [68] can be used in the polybenzoxazine formulation. Bisbenzoxazines have also been prepared through the reaction of mono-phenols and diamines with formaldehyde [77].

### 2.3. Cellulose and Chitosan

Cellulose and chitin are the most important polysaccharides [6]. Bacterial xanthan gum has been utilized as a bio-based resin [78]. However, not all common polysaccharides are useful as biorenewable polymers. Various approaches have been investigated for the preparation of composites consisting of cellulose particles embedded in a cellulose matrix [79]. Invariably, successful composites depend on the solubility of matrix and reinforcement [80], which can be tuned through pretreatment processing [81,82]. Cellulosic matrices have also been reinforced with metal particles for antimicrobial and magnetic applications [83]. Indeed, Fe_2_O_3_ nanoparticles embedded in a sodium carboxymethyl cellulose matrix successfully responded to a magnetic field [84]. These materials may be excellent candidates for biomedical applications due to the reduced risks of bacteria-born infections. The development of these antimicrobial materials may also represent a new direction in the synthesis of bio-based structures with improved biological resistance for increased durability in a specific environment.

There exists a great variety of applications for chitosan [85,86], including self-healing composite anti-corrosion coatings [87], absorbent biopolymer membranes [88,89], and several biomedical uses due to their physical properties, chemical stability, biocompatibility, biodegradability, and ability to undergo chemical modification [90,91,92,93]. More specifically, chitosan has been reinforced with calcium phosphate nanocrystals for tissue engineering with strong adherence to natural bone [94]. Also, chitosan nano- and micro-particles have been used for drug delivery [95,96,97]. Chitosan has also been reinforced with cellulose nanocrystals [98], and nanofibers [99], leading to materials with satisfactory mechanical and water vapor barrier properties for food packaging [99]. The addition of glycerol as a plasticizer to such systems has also been investigated [100,101]. Other reinforcement materials for chitosan-based matrices are multi-walled carbon nanotubes [102], and gold nanoparticles [103].

### 2.4. Polyhydroxyalkanoates (PHAs)

Monomer length in polyhydroxyalkanoates (PHAs) depends on the producing bacteria and molecular weights vary with growth medium conditions. For instance, high molecular weight polyhydroxyoctanoate can be produced in diethylene glycol-rich medium [104]. In tissue engineering, PHA composites have been prepared with hydroxyapatite, bioactive glass, and glass-ceramic fillers [105]. It has been recently shown that the biodegradability of polyhydroxybutyrate (P3HB) and P3HB/valerate copolymers depends greatly on molecular weight, processing conditions, and crystallinity [106].

### 2.5. Proteins

The addition of keratin to synthetic elastomers results in materials with good thermal, mechanical, flame resistant, and thermo-oxidative properties [107]. Similarly, addition of soy protein to petroleum-based latexes results in a material with properties comparable to carbon black-filled elastomers [108]. Investigation of the interaction of vegetable oils and proteins in peanut oil emulsions revealed that protein-coated droplets are stabilized via disulfide crosslinking [109].

## 3. Poly(lactic acid) (PLA) and Related Polymers

**Figure 1:**
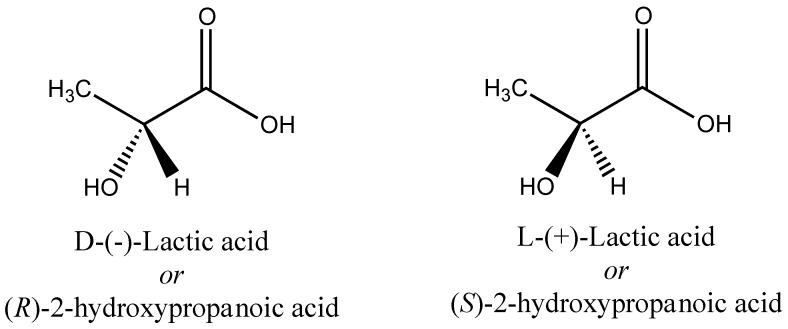
Isomeric forms of lactic acid.

Among the biodegradable polymers, poly(lactic acid) (PLA) is the one of the most important. PLA has been extensively studied for a wide range of applications covering disposable household items, food packaging, agricultural films, drug delivery systems, and implantable biomedical devices [110]. There has been an abundance of literature in recent years related to the production of lactic acid and PLA. There have been recent reviews on lactic acid related to production of lactic acid from lignocellulose [111] production by fermentation processes [112], and lactic acid as a platform chemical for chemical synthesis [113]. For PLA, there have been many reviews of processing technologies [114], with some focused specifically on PLA foaming [115], PLA crystallization [116], and PLA-based nanocomposites [117]. Lactic acid (2-hydroxypropanoic acid) has two optically active configurations lactic acid (l-(+)-lactic acid and d-(−)-lactic acid), as shown in Figure 1 [118]. Both l- and d-enantiomers are produced naturally [119].

PLA is a linear, aliphatic polyester thermoplastic that is produced commercially by ring opening polymerization of lactide [120]. The ratios of l- and d-isomers influence the properties of PLA [116]. High molecular weight polymers are now possible due to advances in processing techniques greatly expanding the range of applications where PLA can be used [121].

### 3.1. Biodegradation of PLA

Biodegradation of plastics is a complex process involving multiple steps and pathways [122]. The initial degradation step is for PLA to be broken down into monomers or low molecular weight oligomers, where the ester bonds are cleaved hydrolytically [123]. Reduction in molecular weight into smaller water soluble fragments is required to facilitate uptake into microorganisms [124]. PLA becomes water soluble when the molecular weight (*M*_n_) is below 20,000 g/mol [124]. The initial degradation into smaller fragments is a rate limiting step in biodegradation processes [125]. After uptake into the microorganisms, subsequent metabolic activity breaks the polymers down into metabolic end products such as carbon dioxide and water, while a portion of the carbon is converted into biomass [126].

As Nampoothiri et al. pointed out, environmental degradation might be an equally appropriate name for the overall mechanism of PLA biodegradation because abiotic and biotic processes occur simultaneously [119]. Environmental composting of PLA proceeds efficiently under adequate conditions due to the combined effects of hydrolysis and microbial activity [127]. Furthermore, elevated temperatures encountered during composting accelerate the hydrolysis process of PLA, especially when temperatures exceed 50 °C [128]. Extracellular enzymes released by specific microorganisms can cause cleavage of PLA chains, which may contribute to the degradation process. However, some studies have shown that extracellular enzymes do not significantly accelerate depolymerization [125].

**Figure 2:**
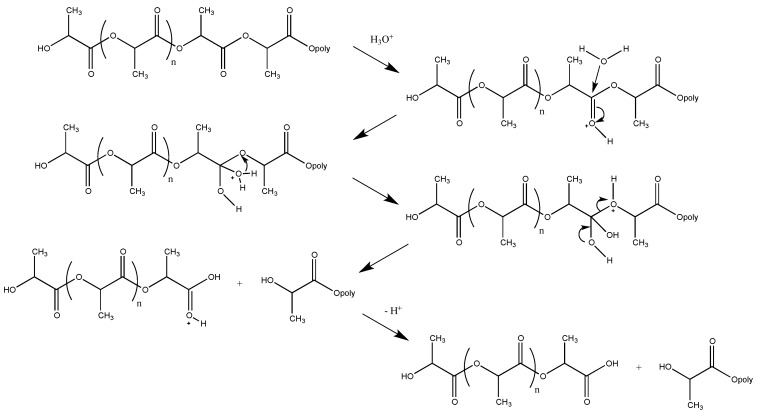
Acid-catalyzed hydrolysis of poly(lactic acid) (PLA).

The reaction mechanism for the hydrolysis of polyesters is bimolecular nucleophilic substitution reaction (*S*_N_2), which may be catalyzed with either acids or bases [129]. A schematic of acid-catalyzed hydrolytic cleavage of PLA chains is shown in Figure 2 [124]. Furthermore, the reaction rates for hydrolytic degradation are dependent on a number of factors including: temperature [130], size and shape of samples [131], molecular weight [129], crystallinity [132], and relative humidity [133].

Degradation of PLA and other aliphatic polyesters biomedical devices inside animal or human tissue is also controlled by hydrolytic mechanisms [134]. Biodegradable devices degrade slowly over time and eventually become absorbed by the body [135]. Many factors affecting hydrolytic degradation of polyesters in the environment (e.g., morphology, crystallinity, sample size, molecular weight) also affect hydrolytic degradation inside of body tissues. However, other factors, like sterilization and site of implantation, can play a role in degradation of biomedical devices [136].

### 3.2. Influence of UV Irradiation on Biodegradation

In recent years, the agricultural industry has been using biodegradable polyesters, including PLA, to make films for mulching applications [137,138]. These mulching films help retain moisture in the soil and modulate the surface temperature [139,140]. It is well known that irradiation of polymers can lead to changes in the polymer structure via different mechanisms including chain scission (e.g., Norrish I or Norrish II) or crosslinking (by recombination of two free radicals) [141]. The impacts of solar radiation on the mechanical properties of PLA have been investigated because mulching films have extended exposure to sunlight. The impacts of irradiation on polymer biodegradability are also a concern, and as a result, there have been studies on polymers to examine the biodegradability after UV irradiation [141].

Polymers that crosslink during irradiation tend to have reduced biodegradation because the higher molecular weight decreases the ability for uptake by microorganisms [142]. Mulching films must be sufficiently biodegradable that they can be tilled into the soil or collected with crop residue for composting without causing ecotoxicity to the soil [143,144]. A recent study observed that PLA degraded by UV irradiation for extended periods of time reduces the rates of biodegradation by microorganisms suggesting the PLA was transformed into poorly assimilated solids [145]. However, a study by Stloukal et al found irradiation of PLA leads to chain scissions rather than crosslinking [138]. Furthermore, this study found, at least for PLA biodegradation, that the specific surface area was a more important factor than the extent of photooxidation [138].

### 3.3. Degradation of PLA Composites and PLA Blends

Pure PLA is difficult to use in most applications due to its brittleness (high modulus of elasticity coupled with low strains at break) [146]. A common strategy for overcoming the brittleness of PLA is blending with other polymers that will improve the overall mechanical properties. Another technique is to add nanofillers derived from clays, carbon, cellulose, or other sources to form PLA-based nanocomposites [117,147]. In certain applications, chemical additives or modified polymers are deliberately added to biodegradable polymers in order to limit the degradation rate of polymers when durability or extended shelf life is needed [148,149]. Since the presence of other polymers and/or nanofillers can impact biodegradation, there have been investigations on biodegradation of PLA nanocomposites and PLA blends.

Polymer blends may have different morphologies based on blending ratios, which will impact biodegradation processes. Furthermore, various biodegradable polymers degrade at different rates [150,151]. PLA/poly(hydroxybutyrate) (PHB) blends, which are promising food packaging materials, have been shown to be compostable under normal compositing conditions [152]. Certain PLA/thermoplastic polyester polyurethane (TPU) blends have also demonstrated biodegradability. The degradation profiles of the PLA/TPU blends were correlated to the different polymer morphologies based on the blend ratios [153]. Poly(butylene-adipate-*co*-terephtalate) (PBAT), an aliphatic-aromatic copolyester that is also biodegradable, has been blended with PLA to increase the toughness of PLA [154,155]. Two recent studies on the biodegradability of a PLA/PBAT blends shows that the blends biodegrade at a slower rate than either PLA or PBAT [151,156].

Biodegradation studies have found that PLA nanocomposite films, containing either native or organo-modified montmorillonites (MMT) nanoclay fillers, show faster biodegradation onset than pure PLA films [123,157]. Another group, Fukushima et al., also found that organically modified fluoro-hectorite increased the degradation rate compared to pure PLA [158,159]. This indicates that PLA composites containing nanoclay fillers may be treated at composting facilities alongside pure PLA.

### 3.4. PLA-Degrading Microorganisms

Many microorganisms have been identified that will biodegrade aliphatic polyesters, such as poly(β-hydroxyalkanoate), poly(ε-caprolactone), poly(hexamethylene carbonate), and poly(tetramethylene succinate) [160]. However, isolating microorganisms that biodegrade PLA has been more difficult. In 1997, Pranamuda et al reported the first PLA degrader, *Amycolatopsis* strain HT-32, which was isolated from soil [161]. According to an earlier review by Tokiwa and Calabia, most PLA-degrading microorganisms are bacteria, belonging to related genera within the taxonomic family of *Pseudonocardiaceae*, including *Amycolatopsis*, *Lentzea*, *Kibdelosporangium*, *Streptoalloteichus*, and *Saccharothrix* [162]. In 2001, *Tritirachium album* was the first isolated fungal strain reported to degrade PLA [163].

Ongoing research continues to isolate previously unknown PLA degraders belonging to other families and genera. A new strain of mesophilic bacterium, *Stenotrophomonas maltophilia* LB 2–3, was isolated from compost on a pear-tree farm in South Korea [145]. Previously unreported strains of *Rhizobium* sp. and *Alpha proteobacterium* found in agricultural soils from Vietnam were able to biodegrade PLA/ethylene vinyl acetate (EVA) [146]. Recently, a study reported the isolation of a PLA-degrading bacteria (*Thermopolyspora flexuosa*, *or* FTPLA) [125].

### 3.5. Methods of Monitoring Biodegradation

In recent years, there have been many efforts to standardize methods of measuring polymer biodegradation, which has resulted in the publication of various standards and protocols by the International Organization for Standardization (ISO), American Society for Testing and Materials (ASTM International), governmental agencies, and other professional organizations [164,165]. Lucas et al. wrote a comprehensive review of the mechanisms and estimation techniques of polymer biodegradation [166]. Kale et al. provided an excellent summary of the standards related to composting of plastics [167]. Specific standards are used to measure biodegradability under different environmental conditions like composting, anaerobic digestion, or waste water treatment [20]. The European standard for compostability of packaging and packaging waste material, European Norm EN13432:2000, has the strictest requirements for evaluating biodegradability and compostability [168]. Two other important standards on compostablity are ASTM D 6400 and BNQ (Bureau de normalisation du Québec) 9011-911 which are used in the USA and in Canada, respectively. Laboratory techniques for monitoring biodegradation include measurement of evolved CO_2_, weight loss, changes in molecular weight, changes in mechanical properties, and radiolabeling [151,169].

## 4. Other Bio-Based Polymers with Potential for Biodegradability

Over the past decades there has been an increased social and economic demand for alternative energy, materials, and resources to replace current nonrenewable, fossil fuel-based products [170]. With the constant fluctuation in petroleum prices, it is imperative to find alternative resources in order to leverage the uncertainty of the oil market. Bio-based polymers derived from renewable feedstocks are a strategic area of sustainable development [171]. One key element of bio-based polymer research is biodegradability [172], although not all bio-based polymers are inherently biodegradable. As explained previously, biodegradation takes place through a reaction of enzymatic and/or chemical deterioration associated with living organisms [19]. Therefore, some petroleum-based products can be biodegradable, such as poly(caprolactone) (PCL) [19]. In this section, only polymers that are concomitantly biodegradable and bio-based will be discussed.

Several factors make bio-based polymers and composites attractive for environmentally friendly research, including their potential for biodegradability, conservation of petroleum demands, accessibility, low toxicity, economic efficiency, and low carbon footprint [173]. Bio-based polymers are macromolecules derived from plants, trees, bacteria, algae, or other bio-renewable resources. They are often degradable through microbial processes such as composting. Some of the most common natural biopolymers include cellulose, proteins, starches, and polyesters. Their widespread use is limited due to an intrinsic difficulty in ensuring reproducibility of the main properties [174]. For example, although several distinct microorganisms are able to produce polyhydroxyalkanoates (PHAs), their polymer composition differs depending on the microorganism’s nourishment intake.

In order to obtain better control over polymer property reproducibility, bio-based monomers or resins have been isolated, modified, and/or synthesized. Polyurethanes, polyester amides, polyolefins [175], and alkyd resins [176] are some of the bio-based resins currently available at a commercial scale. In the past, in order to improve their mechanical properties, these polymers have been reinforced with inorganic and organic materials [175], while improvement of their processability has been accomplished, in some cases, by the use of volatile organic solvents [176]. For the sake of text conciseness, this section will be limited to bio-based and biodegradable polymers currently used in the areas of medical applications, plastics, elastomers, adhesives, bio-based compatibilizers and additives.

### 4.1. Medical Applications

Polymers have been a valuable material in medicine, and over the past half-century, biodegradable, biocompatible polymers have gained increasing popularity in drug delivery. Indeed, bio-based polymers that are capable of dissolving in the body after the drug is delivered to its target are highly desirable [177]. It is possible to design polymers for specific applications by manipulation and control of the polymer composition, thermal behavior, hydrophobicity/hydrophilicity, mechanical properties, ability to retain the encapsulated or entrapped drug, and the interactions of the polymer in a biological environment [178]. Other applications outside the realm of drug delivery include surgical devices, implants, tissue engineering, gene therapy, regenerative medicine, coatings on implant biosensors, components of diagnostic assays, bio-adhesives, ocular devices, and materials for orthopedic applications [178,179].

The applications of a specific bio-based polymer are greatly dependent on its composition. Unsaturated oils have become an attractive source for polymers due to their carbon-carbon double bonds within the fatty acid chains. These double bonds serve as ideal reactive sites for polymerization [180]. Hydrophobicity and flexibility of vegetable oil-based polymers can be tuned by monomer composition and by the selection of the specific oil to be used as a monomer. It has been shown that oil-based polymers do not form uniform blends due to the variable fatty acid composition within each oil, which can result in a micro-phase separation of the matrix, compromising the mechanical properties of the final polymeric material [178,181]. In order to limit this effect, monomeric triglyceride units have been added to a filler or a template backbone polymer, such as polyanhydrides, co-polyesters, or polyamides. In anticancer treatments, fatty acid dimer (FAD) systems were originally created to control the release of water soluble and unstable chemotherapeutics [182]. Drugs impregnated with FAD were shown to allow for prolonged and controlled drug release [182]. FADs have also been shown to have local anesthetic and antibiotic properties. Most fatty acids undergo condensation to form polyanhydrides that can easily be hydrolytically degraded. Though polyanhydrides can degrade by surface erosion, there are many factors that influence the mechanism and rate of degradation [178].

**Figure 3:**
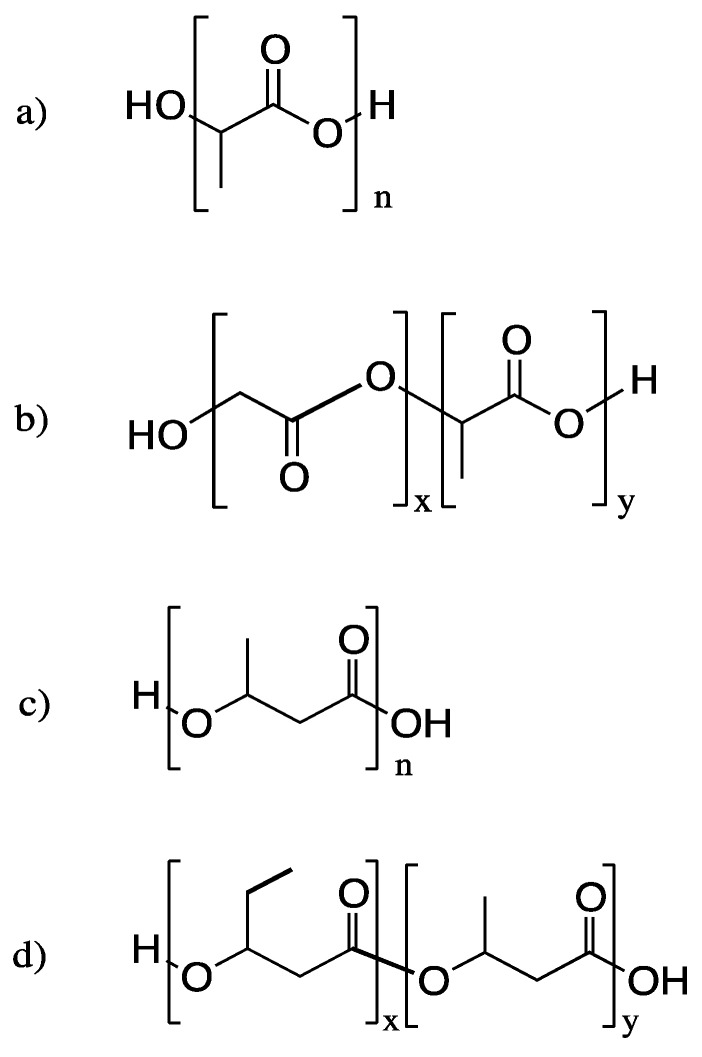
Chemical structures of (**a**) poly(lactic acid) (PLA); (**b**) poly(lactic-*co*-glycolic acid) (PLGA); (**c**) poly-3-hydroxybutyrate (or poly-hydroxybutyric acid, PHB); (**d**) poly(3-hydroxybutyrate-*co*-3-hydroxyvalerate) (PHBV).

Tissue engineering is a popular field in medicine and the current market is estimated to be approximately $23 million dollars, with a projected continuous growth in the coming years [183]. Polyesters are one of the most competitive polymers for regenerative implantation surgeries, therapeutic cell culturing, and tissue repair. Of all the current commercial products, polyesters act as biologically passive supporting materials such as sutures, surgical mesh or netting, or drug-releasing vehicles. To address more advanced medical and regenerative applications, polyesters are modified to overcome issues such as low cell adhesion, hydrophobicity, and inflammatory side effects. Some of the most commonly used bio-based polyesters are poly(lactic acid) (PLA), poly(lactic-*co*-glycolic acid) (PLGA), poly-3-hydroxybutyrate (or poly-hydroxybutyric acid, PHB), poly(3-hydroxybutyrate-*co*-3-hydroxyvalerate) (PHBV) (Figure 3) [19,184,185]. PLA is most commonly used in sutures due to its high tensile strength and elongation at break [186].

### 4.2. Plastics

Plastics play an important role in our society. They are used in food packaging, clothing, construction, communication, transportation, and health care equipment and supplies [185]. Among the currently available bioplastics, PLA, starch, and PHAs are the most attractive to study because they can be processed with conventional methods, have comparable physical properties, and can be produced in an economically efficient fashion, in large scale quantities [187]. In the realm of plastics, PLA, a derivative of starch, is very popular due to its virtually neutral carbon footprint. Indeed, atmospheric carbon dioxide is consumed by plants during photosynthesis, compensating for CO_2_ discharge during combustion or biodegradation [188].

**Figure 4:**
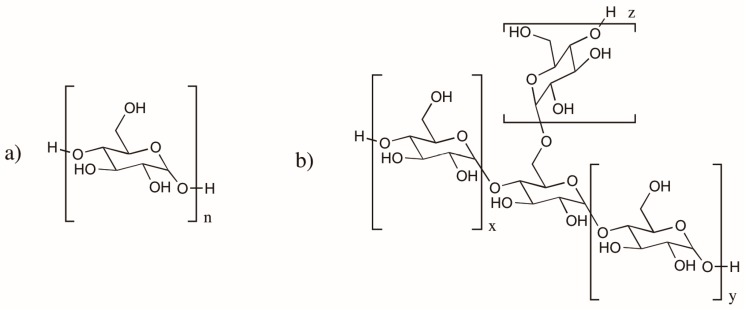
Chemical structure of (**a**) amylose; and (**b**) amylopectin.

Starch is one of the most abundant natural polymers extracted from agricultural sources, such as corn, wheat, potato, and cassava. It is composed of repeating glucose monomers, and is found in its linear form as amylose, and in a branched form as amylopectin (Figure 4) [187,189]. The main applications of starch include use as a thickener-stabilizer and gelling agent in foods. Gelatinized starch and processed starch are utilized in the textile industry and papermaking industry. Starch can only be used as a plastic, without the need of a second film-forming substance, upon addition of plasticizers. Starch can only be dissolved in ionic liquids and some organic solvents, and undergoes gelatinization when mixed with hot water. The human body naturally contains enzymes that break it down. Starch is capable of melt-forming when mixed with glycerol. It is therefore used as a capsule material in applications such as food trays. Despite the low compatibility of starch with some biodegradable polymers [190], blends of starch and biodegradable plastics have been developed and are marketed as film, foamed plastic, and coatings [188].

Similar to PLA, PHA is associated to a carbon-neutral footprint, meaning that degradation of the polymer does not correspond to an overall increase of atmospheric CO_2_ levels. PHA is produced by microorganisms, and is therefore bio-based. PHA is also biodegradable through enzymatic activity and is known for its ultraviolet radiation protection capabilities in the organisms that produce it [191]. PHA can be extracted from its producing bacteria and processed through extrusion for production of rigid and flexible plastics for various biomedical applications. PHA can also be considered for applications including packaging, paper coatings, non-woven fabrics, adhesives, films, and performance additives. PHAs are gaining attention due to their promising biodegradable properties in different environments other than composting plants [192].

### 4.3. Adhesives and Elastomers

Pressure sensitive adhesives (PSA) are commercially known as adhesive tape. They are permanently tacky at room temperature and should adhere to various surfaces with light pressure. Common PSAs are adhesive tape, postage stamps, labels, and duct tape. PSAs need to have good flexibility, tack, and peel strength. Most are composed of petroleum-based acrylate polymers, and are formulated with two components, an elastomer and a tackifier. Rosin derivatives can be used as a bio-based tackifier and natural rubber may be used as the elastomer component. Although finding a completely bio-based and biodegradable adhesive that is cohesive may be difficult, studies have shown that epoxidized soybean oil, polyethylene glycol, and PHA satisfy the Dahlquist criterion for elastomer use. PSAs are generally considered for single-use only, and end up creating a significant amount of waste. Creating a PSA system that is bio-based and biodegradable could help alleviate the waste production and should be further investigated [193].

### 4.4. Compatibilizers, Biocomposites and Biofibers

**Figure 5:**
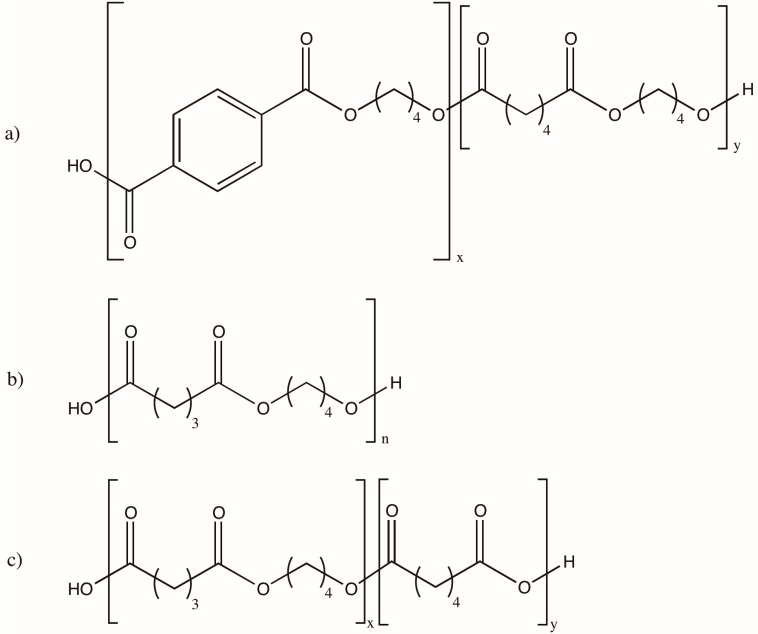
Chemical structure of (**a**) poly(butylene-adipate-*co*-terephtalate) (PBAT); (**b**) poly(butylene succinate) (PBS); and (**c**) poly(butylene succinate-*co*-adipate) (PBSA).

Despite their great positive environmental impact, most bio-based and biodegradable polymers’ mechanical properties are inferior to their petroleum-based counterparts. Compatibilizers and/or reinforcing agents, such as inorganic fillers and fibers [176], are often added to bio-based polymers in order to improve their mechanical properties and make them suitable for structural applications. For instance, the non-renewable, biodegradable polymer, poly(butylene adipate-*co*-terephthalate) (PBAT) [194], has been blended with PLA in order to confer flexibility and a higher elongation at break in comparison to other biodegradable polymers. The blend, however, exhibits poor thermal and mechanical properties. Compatibilizers that are of considerable toughness, such as poly(butylene succinate) (PBS), PHBV and the non-renewable, biodegradable polymer poly(butylene succinate-*co*-adipate) (PBSA) [194] were added to a PLA/PBAT blend, resulting in a slight decrease of thermal properties and an increase in melt flow with PBS, while no change was observed with PBSA or PHBV [195]. The chemical structures of PBAT, PBS, and PBSA are provided in Figure 5.

Triglycerides and epoxidized oils have been investigated as compatibilizers with PLA because of their ester or epoxy groups, which can be degraded by microorganisms. Some oils also contain fatty acids with carbon–carbon double bonds that can act as reactive sites for crosslinking, creating a stronger polymer network. Recent studies have tested the mixture of deodorization oil condensates with PLA and report an increase in ductility in comparison to pure PLA. Deodorization oil condensates are mixtures of different molecules found in vegetable oils [196]. These molecules are recovered via a vacuum steam distillation process, in which steam is passed through the vegetable oil at a very low pressure and high temperature [196].

Bio-fibers or reinforcements have also become increasingly popular as low cost and renewable reinforcing agents. Two common bio-based reinforcing agents are lignin and cellulose. Lignin is more sensitive to light degradation than cellulose. Cellulose is more sensitive to heat degradation than lignin. These factors can affect the choice of bio-based reinforcement [197]. Generally, these biopolymers are highly hydrophilic, but they are typically added to hydrophobic polymer matrices, compromising reinforcement and matrix adhesion. In order to improve reinforcement-matrix adhesion, cellulose treatment that decreases hydrophilicity has been proposed. Grafting techniques have also become popular and can be categorized into three groups, namely grafting of fiber with a single monomer, grafting with a mixture of two or more monomers, and grafting with the polymer directly. Along these lines, bio-based and biodegradable PHBV oligomers have been prepared by transesterification, and PHBV-graft-ethyl cellulose copolymers have been synthesized using 1,6-hexamethylene diisocyanate (HDI) as a grafting agent. In comparison with neat PHBV, the crystallinity of the grafted copolymer decreased and the moisture resistance was improved [198].

### 4.5. Chemical Structure Influence on Biodegradation

There are several studies in the literature suggesting relationships between the chemical structure of substrates and their degradation rates. In one such study, it was shown that the bulkiness of the alkyl chain in nonylphenols has a negative impact on the degradation rate [199]. Indeed, the following order of decreasing degradation rate was observed based on the α-substituents found in nonylphenols: α-dimethyl > α-ethyl-α-methyl > α-methyl-α-npropyl > α-iso-propyl-α-methyl [199]. When evaluating the properties and biodegradability of polymers, a series of copolymers of poly(propylene 1,4-cyclohexanedicarboxylate) and *neo*-pentyl glycol (NPG) was prepared and a correlation between mol % of NPG and mechanical properties was established [200]. Likewise, it was shown that the polymers exhibiting the lowest mechanical properties were the ones with the highest degradation rates [200]. Along the same lines, a series of copolymers of ethylene and propylene oxides were evaluated for their biodegradability and the results indicated that high biodegradability rates depend primarily on (1) the presence of terminal hydroxyl or acyl functional groups to allow for metabolic processes; (2) lower molecular weights; and (3) higher contents of ethylene oxide units [201]. Such findings suggest that in many cases biodegradability may be directed by physico-chemical phenomena, such as the mobility of substrate, its availability, mixing, mechanical properties impacting breaking down of the macrostructure, and/or hydrophilicity of chemical species.

## 5. Conclusions

In conclusion, the importance of the search for more sustainable methods, including biodegradability studies of bio-based polymeric materials has been highlighted. The recent literature on the most significant bio-based polymer systems has been briefly covered with a special emphasis on vegetable oil-based resins and PLA due to their current widespread industrial use and relevance. It has been pointed out, and examples have been presented showing, that bio-based or bio-renewable monomers can lead to materials that may not necessarily be fully or significantly biodegradable, hence the relevance of the current review. It has also been mentioned that the biodegradability of a large number of novel bio-based systems has not been investigated yet. The discussion revolved around the idea that biodegradability consists in the breakdown of matter as the result of the activity of microorganisms that secrete enzymes, which cleave specific chemical bonds or perform very specific chemical reactions. Changes in the functional groups of bio-based monomers, crosslink density, and co-polymerization with non-biodegradable co-monomers can lead to materials that exhibit various degrees of biodegradability, with varying environmental impact.

As rule of thumb, in order for polymers to be biodegradable, they must have a carbon backbone. The degradation process may occur through hydrolytic or enzymatic processes into oligomer units and eventually into monomer units. Fungi, bacteria, and algae are some of the most important organisms in the degradation process. For natural polymers such as polysaccharides or proteins, the polymer is degraded in a biological system by enzymatic processes. The rate of degradation is highly dependent on the chemical structure. Medical applications can significantly benefit from bio-based, biodegradable polymers’ biocompatibility and biodegradability without toxic effects. Other fields where bio-based and biodegradable polymers are becoming increasingly popular are plastics, adhesives, and elastomers. Despite all the recent progress made on bio-based and biodegradable polymers, further advancements are needed before petroleum-based products can be completely replaced.
